# Fibroblast growth factor receptor 2 expression, but not its genetic amplification, is associated with tumor growth and worse survival in esophagogastric junction adenocarcinoma

**DOI:** 10.18632/oncotarget.7782

**Published:** 2016-02-27

**Authors:** Ryuma Tokunaga, Yu Imamura, Kenichi Nakamura, Takatsugu Ishimoto, Shigeki Nakagawa, Keisuke Miyake, Yu Nakaji, Yasuo Tsuda, Masaaki Iwatsuki, Yoshifumi Baba, Yasuo Sakamoto, Yuji Miyamoto, Hiroshi Saeki, Naoya Yoshida, Eiji Oki, Masayuki Watanabe, Yoshinao Oda, Adam J. Bass, Yoshihiko Maehara, Hideo Baba

**Affiliations:** ^1^ Department of Gastroenterological Surgery, Graduate School of Medical Sciences, Kumamoto University, Kumamoto, Japan; ^2^ Department of Surgery and Sciences, Graduate School of Medical Sciences, Kyushu University, Fukuoka, Japan; ^3^ Department of Anatomic Pathology, Pathological Sciences, Graduate School of Medical Sciences, Kyushu University, Fukuoka, Japan; ^4^ Department of Medical Oncology, Dana-Farber Cancer Institute, Boston, Massachusetts, USA; ^5^ Broad Institute of MIT and Harvard, Cambridge, Massachusetts, USA; ^6^ Department of Medicine, Brigham and Women's Hospital and Harvard Medical School, Boston, Massachusetts, USA

**Keywords:** esophagogastric junction adenocarcinoma, esophageal adenocarcinoma, FGFR2, targeting therapy, amplification

## Abstract

**Background:**

Fibroblast growth factor receptor 2 (FGFR2) genetic alterations lead to tumor cell proliferation in various types of cancer. We hypothesized that *FGFR2* amplification is associated with FGFR2 expression, resulting in tumor growth and poorer outcome in esophagogastric junction (EGJ) adenocarcinoma.

**Patients and Methods:**

A total of 176 consecutive chemo-naive patients with EGJ adenocarcinoma were enrolled from two academic institutions. *FGFR2* amplification was examined by real-time PCR (*N* = 140) and FGFR2 expression with immunohistochemical staining (*N* = 176), and compared against clinicopathological factors and patient outcomes. The effects of FGFR2 inhibition or overexpression on cell proliferation, cell cycle, and apoptosis assays were investigated in EGJ adenocarcinoma cell lines. Downstream FGFR2, AKT and ERK were also examined.

**Results:**

Based on the correlation between *FGFR2* levels and FGFR2 overexpression *in vitro*, *FGFR2* amplification was defined as copy number > 3.0. In clinical samples, *FGFR2* amplification and FGFR2 IHC expression were 15% and 61%, respectively. Although these two statuses were significantly correlated (*P* < 0.05), only FGFR2 IHC expression was significantly associated with tumor depth (multivariate *P* < 0.001) and overall survival of patients (univariate *P* = 0.007). Supporting these findings, FGFR2 overexpression was associated with tumor cell proliferation, cell cycle progression, and anti-apoptosis. Selective inhibition of FGFR2 sufficiently suppressed tumor cell proliferation through de-phosphorylation of AKT and ERK.

**Conclusions:**

*FGFR2* amplification was significantly associated with FGFR2 expression. FGFR2 expression (but not *FGFR2* amplification) was associated with tumor growth and patient outcomes. Our findings support FGFR2 as a novel therapeutic target for EGJ adenocarcinoma.

## INTRODUCTION

Esophagogastric junction (EGJ) adenocarcinoma has rapidly increased worldwide [1–3] along with its risk factors; namely, obesity and gastro-esophageal reflux disease (GERD) [4, 5]. The prognosis of EGJ tumors remains poor [6], despite the development of multidisciplinary treatments using cytotoxic agents [7, 8]. Molecular-targeted therapy is an attractive option in the management of gastrointestinal cancer. Trastuzumab, a monoclonal antibody that interferes with HER2/neu, is effective against EGJ adenocarcinoma when combined with chemotherapy [9]. Recently, ramucirumab has emerged as an effective monoclonal antibody targeting VEGFR-2 [10, 11]. However, further molecular targets are necessary to improve the survival rates of this cancer.

Recently, next-generation sequencing technologies have revealed frequent oncogenic amplification as an important genetic alteration in the carcinogenesis of esophageal adenocarcinomas, including EGJ adenocarcinoma [12–15]. We previously identified *FGFR2* amplification in esophageal adenocarcinoma [15]. FGFR2 exerts an oncogenic effect when stimulated by fibroblast growth factors (FGFs) or *FGFR2* alterations [16, 17]. FGFR2 is highly expressed in pancreatic and colorectal cancer, leading to cell proliferation [18, 19]. Based on this evidence, we hypothesized that *FGFR2* amplification is associated with FGFR2 expression, resulting in aggressive tumors and poorer patient outcomes. We also examined FGFR2 as a potentially therapeutic target for EGJ adenocarcinoma.

Therefore, we here investigate the relationships between *FGFR2* amplification and FGFR2 expression, and examine whether FGFR2 expression plays an oncogenic role in EGJ adenocarcinoma. For this purpose, we accessed a database of 176 patients with EGJ adenocarcinoma. Our findings suggest that FGFR2 can be a substantial therapeutic target as well as a biomarker for FGFR targeting therapy, in EGJ adenocarcinoma.

## RESULTS

### The associations among FGFR2 amplification, FGFR2 mRNA, and FGFR2 expression in EGJ adenocarcinoma cell lines

**Figure 1:**
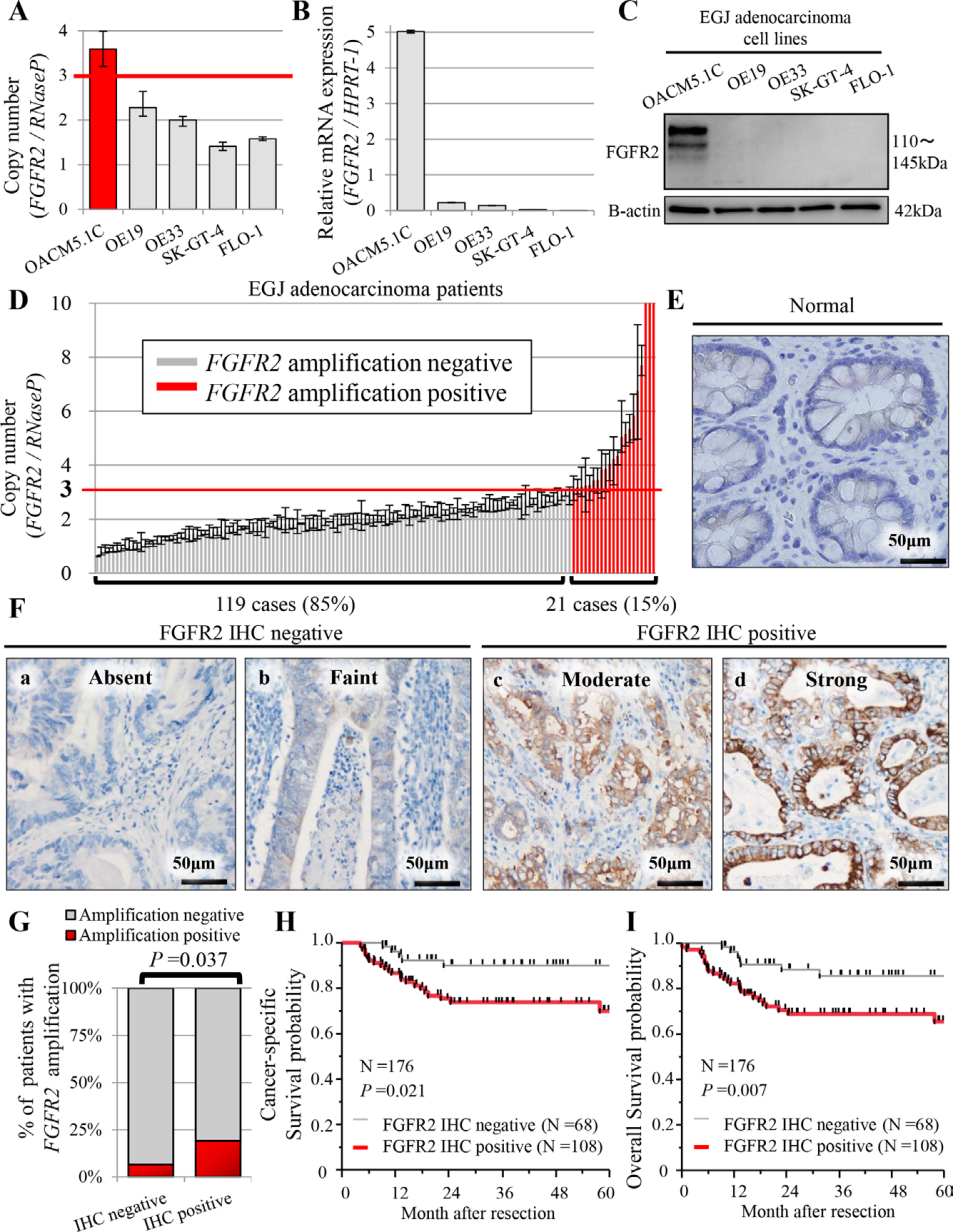
Profiles of FGFR2 status in five human EGJ adenocarcinoma cell lines (A–C), and in patients with EGJ adenocarcinoma (*N* = 176) (D–I) (**A**) *FGFR2* copy number obtained in real-time PCR assay; (**B**) mRNA expression by qRT-PCR assay; (**C**) FGFR2 expression by western blot analysis; (**D**) Distributions of *FGFR2* copy number (*N* = 140). For *FGFR2* amplification, the copy number gain must exceed 3.0 copies. *FGFR2* amplification was observed in 21 cases (21/140 = 15%); (**E**) FGFR2 was not expressed in normal glandular epithelium; (**F**) (**a, b**) Cases with absent or faint FGFR2 staining were assessed as FGFR2 IHC-negative; (**c, d**) cases with moderate or strong FGFR2 staining were FGFR2 IHC-positive; (**G**) Association between FGFR2 IHC positivity and *FGFR2* amplification; (**H**) Cancer-specific survival in positive and negative FGFR2 IHC cases; (**I**) Overall survival in positive and negative FGFR2 IHC cases.

We examined whether *FGFR2* amplification correlates with *FGFR2* mRNA and FGFR2 expression in the five kinds of human EGJ adenocarcinoma cell lines, namely OACM5.1C, OE19, OE33, SK-GT-4 and FLO-1. The correlation was found in three of the cell lines (OACM5.1C, SK-GT-4, and FLO-1) (Figure 1A–1C), but not in OE19 and OE33.

The *FGFR2* copy number exceeded 2 in OACM5.1C (copy number 3.59 ± 0.39) and OE19 (copy number 2.28 ± 0.27). In OACM5.1C cells only, *FGFR2* amplification was correlated with high FGFR2 expression and with *FGFR2* mRNA (Figure 1A–1C). Although the *FGFR2* copy number was relatively high in the OE19 cell line, it was not associated with *FGFR2* mRNA or FGFR2 expression (Figure 1A–1C). Based on these findings, we set the cut-off value for *FGFR2* amplification as 3.0. In the other cell lines, the *FGFR2* copy number was 2 or lower; namely, OE33 (copy number 2.00 ± 0.11), SK-GT-4 (copy number 1.41 ± 0.09), and FLO-1 (copy number 1.58 ± 0.04), correlating with low levels of *FGFR2* mRNA and FGFR2 expression (Figure 1A–1C). Therefore, for investigating the oncogenic effect of FGFR2 in EGJ adenocarcinoma cell lines, we adopted OACM 5.1C and FLO-1 as high and low expressers of FGFR2, respectively.

### Clinicopathological factors and statuses of FGFR2 amplification and FGFR2 IHC

**Figure 2:**
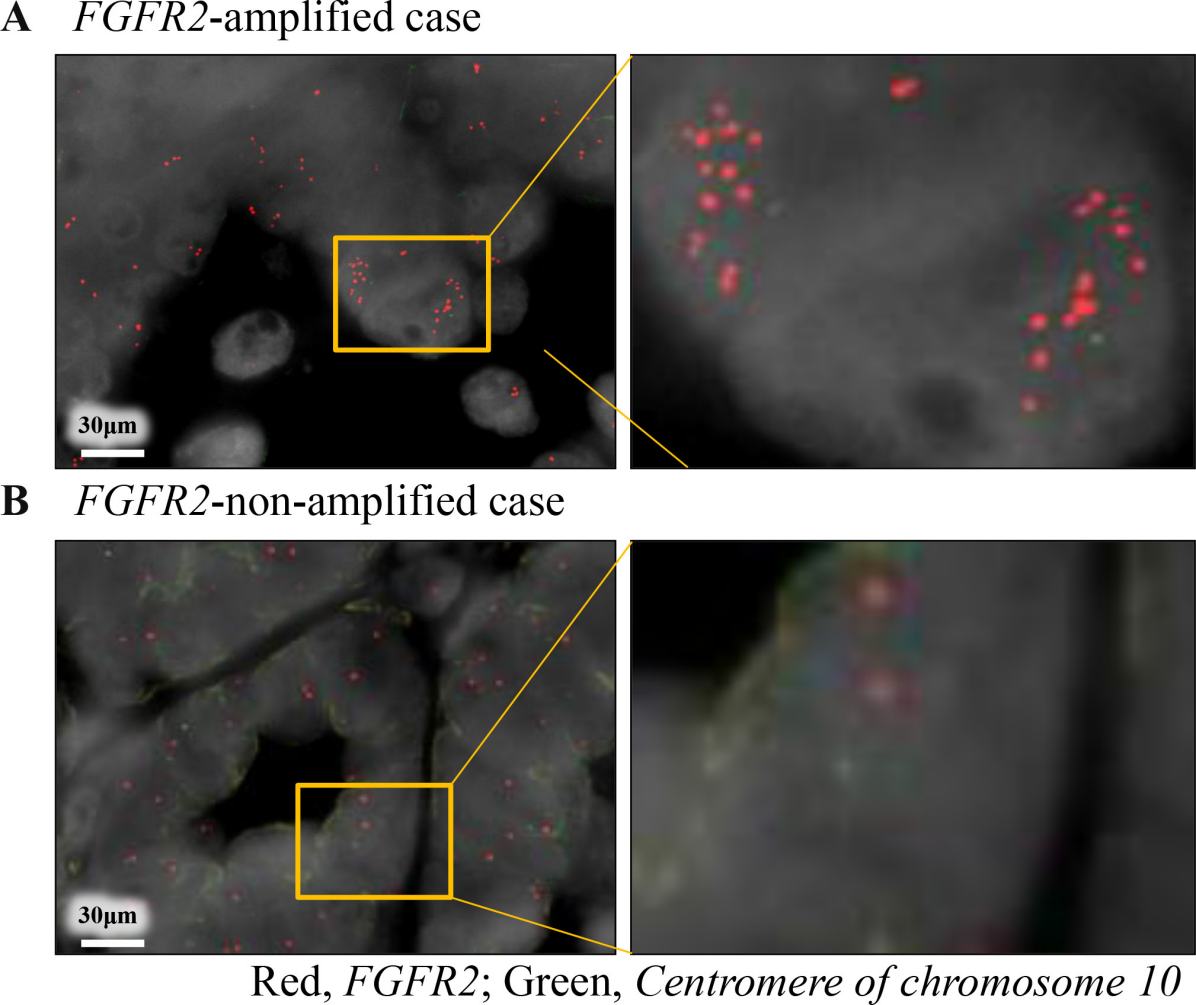
Dual-color fluorescence *in situ* hybridization coincided with the results of copy number assay by real-time PCR reaction Red and green signals indicate *FGFR2* gene and *centromere of chromosome 10* probes, respectively. (**A**) FGFR2-amplified case obtained by real-time PCR reaction; (**B**) FGFR2-non-amplified case obtained by real-time PCR reaction.

We successfully extracted the tumor DNA from 145 cases with sufficient tumor area on the slides (145/176 = 82%), and assayed the *FGFR2* copy number in 140 cases (140/145 = 97%) (Supplementary Figure 1). Twenty-one (21/140 = 15%) of these assays tested positive for amplification (> 3.0 copies, Figure 1D, Table 1). *FGFR2* amplification by real-time PCR based method was concordant with that by FISH, in the two selected cases (Figure 2). *FGFR2* amplification was not associated with any clinicopathological factors (Table 1).

**Table 1:** Associations between FGFR2 positivity and clinicopathological factors in EGJ adenocarcinoma patients with tumor resection

	*FGFR2* amplification			FGFR2 IHC		
	Negative	Positive	*P* value	Negative	Positive	*P* value
**No. patients (%)**	119 (85%)	21 (15%)		68 (39%)	108 (61%)	
**Age**			0.971			0.520
Mean ± SD	68 ± 12	67 ± 12		67 ± 11	69 ± 12	
**Sex**			1.000			0.308
Male	96 (81%)	17 (81%)		51 (75%)	88 (81%)	
Female	23 (19%)	4 (19%)		17 (25%)	20 (19%)	
**Siewert classification**			0.945			0.025
I	20 (17%)	4 (19%)		5 (7%)	24 (22%)	
II	25 (21%)	4 (19%)		18 (27%)	23 (21%)	
III	74 (62%)	13 (62%)		45 (66%)	61 (57%)	
**Tumor depth**			0.689			< 0.001
T1	37 (31%)	6 (29%)		42 (62%)	23 (21%)	
T2	19 (16%)	2 (9%)		13 (19%)	10 (9%)	
T3	46 (39%)	8 (38%)		9 (13%)	52 (48%)	
T4	17 (14%)	5 (24%)		4 (6%)	23 (22%)	
**Tumor size (mm**)			0.919			0.110
Mean ± SD	54 ± 7	56 ± 16		45 ± 10	67 ± 8	
**Lymph node metastasis**			0.286			< 0.001
Negative	66 (55%)	9 (43%)		53 (78%)	49 (45%)	
Positive	53 (45%)	12 (57%)		15 (22%)	59 (55%)	
**Distant metastasis**			0.990			0.030
Negative	108 (91%)	19 (90%)		66 (97%)	94 (87%)	
Positive	11 (9%)	2 (10%)		2 (3%)	14 (13%)	
**Histopathological types**			0.305			0.235
Well-moderate	80 (67%)	17 (81%)		52 (76%)	73 (68%)	
Poorly	39 (33%)	4 (19%)		16 (24%)	35 (32%)	

In multiple-hypothesis testing, the significant *P* value was adjusted to *P* = 0.05/16 = 0.003. Thus, a *P* value between 0.05 and 0.003 should be regarded as borderline significant.

*EGJ*, esophagogastric junction; *FGFR2*, fibroblast growth factor receptor 2; *IHC*, immunohistochemistry; *SD*, standard deviation. *N* = 176.

FGFR2 expression was investigated by IHC staining of all cases (*N* = 176). FGFR2 was expressed heterogeneously in the membranes of tumor cells, but not in that of normal epithelial cells (Figure 1E, 1F, Supplementary Figure 2). The FGFR2 IHC assay was positive in 108 cases (108/176 = 61%), and significantly associated with FGFR2 amplification (*P* = 0.037) (Figure 1G). In 108 FGFR2-positive cases, the mean percentage of FGFR2-positivity was 57 ± 22% of the tumor (range, 5% to 100%), indicating heterogeneous FGFR2 expression in EGJ adenocarcinoma (Supplementary Figure 2). In 21 cases with FGFR2 amplification, 16 cases (16/21 = 76.2%) were positive for FGFR2 IHC. FGFR2 IHC positivity was also significantly associated with tumor depth and lymph node metastasis (all *P* < 0.001, Table 1) [Bonferroni-corrected *P* < 0.003 (= 0.05/16)]. Notably, tumor depth was significantly related to FGFR2 IHC positivity in the multivariate logistic analysis (multivariate odds ratio = 4.57; 95% confidence interval 1.99-11.02; *P* < 0.001, Table 2) [Bonferroni-corrected *P* < 0.004 (= 0.05/14)]. There was no association between FGFR2 amplification status and clinicopathological factors (Supplementary Table 2).

**Table 2:** Univariate and multivariate logistic analysis of FGFR2 IHC status in EGJ adenocarcinoma patients with tumor resection

Factors		FGFR2 IHC positive status (%)	Univariate analysis				Multivariate analysis		
			OR	95% CI	*P* value	OR	95% CI	*P* value
**Age**	≥70/<70	56 (65%)/52 (57%)	1.36	0.74-2.52	0.317	1.05	0.50-2.15	0.904
**Sex**	Male/female	88 (63%)/20 (54%)	1.47	0.70-3.05	0.308	1.10	0.47-2.54	0.830
**Siewert classification**	I-II/III	47 (67%)/61 (58%)	1.51	0.81-2.86	0.199	1.98	0.96-4.25	0.065
**Tumor depth**	T2-T4/T1	85 (77%)/23 (35%)	5.97	3.09-11.88	<0.001	4.57	1.99-11.02	<0.001
**Lymph node metastasis**	Positive/negative	59 (80%)/49 (48%)	4.25	2.18-8.68	< 0.001	1.88	0.82-4.38	0.137
**Distant metastasis**	Positive/negative	14 (88%)/94 (59%)	4.91	1.32-31.96	0.015	2.28	0.55-15.57	0.275
**Histopathological type**	Poorly/well-moderate	35 (69%)/73 (58%)	1.56	0.79-3.17	0.202	1.14	0.49-2.67	0.763

In multiple-hypothesis testing, the significant *P*-value was adjusted to *P* = 0.05/14 = 0.004. Thus, a *P* value between 0.05 and 0.004 should be regarded as borderline significant.

*CI*, confidence interval; *EGJ*, esophagogastric junction; *FGFR2*, fibroblast growth factor receptor *2;* OR, odds ratio. *N* = 176.

### FGFR2 amplification status, FGFR2 IHC status, and patient outcome

The 5-year CSS probabilities were 81.7% and 81.1% in cases testing positive and negative for *FGFR2* amplification, respectively. The respective 5-year OS probabilities were 71.4% and 75.9%. *FGFR2* amplification status was not associated with patient outcome (Supplementary Figure 3).

The 5-year CSS probabilities were 70.1% and 90.2% in FGFR2 IHC-positive and negative cases, respectively, with corresponding 5-year OS probabilities of 65.7% and 85.8%. The CSS was poorer in patients with FGFR2 IHC-positive tumors, but the significance was borderline (*P* =0.021, Figure 1H). The OS, however, was considerably worse in patients with positive FGFR2 IHC tumors than in their negative-testing counterparts (*P* = 0.007; significant at the *P* = 0.0125 (0.05/4) level; Figure 1I). In the survival analysis according to combinations of *FGFR2* amplification and FGFR2 expression, the patients with *FGFR2*-amplified/FGFR2-IHC-positive tumor seemed to experience worse outcomes compared to the other groups, but there was no significant difference (Supplementary Figure 4).

### Proliferation, cell cycle and apoptosis assays of FGFR2 knockdown cell line

**Figure 3:**
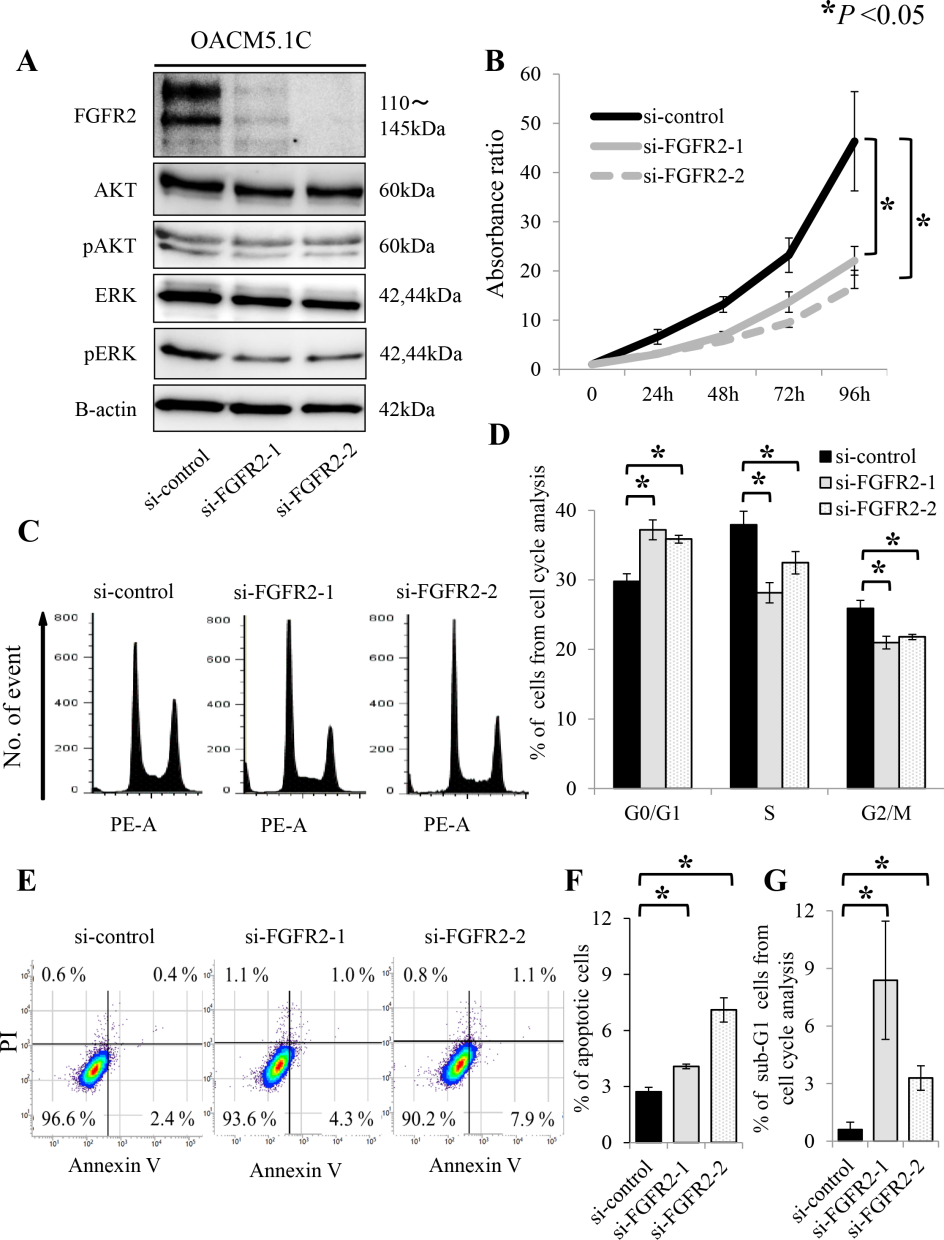
FGFR2 knockdown induces de-phosphorylation of AKT and ERK, and suppresses cell proliferation, through anti-apoptosis and cell cycle arrest in the FGFR2-expressing cell line OACM5.1C (**A**) De-phosphorylation of AKT and ERK after *FGFR2* knockdown by siRNAs targeting *FGFR2* (si-FGFR2); (**B**) Cell proliferation after transfection with si-control or si-FGFR2s; (**C**) Distributions of cell cycle populations; (**D**) Proportions (%) of G0/G1, S, and G2/M cells in the cell cycle distribution; (**E**) Distributions of apoptotic cells; (**F**) Apoptotic cells, identified as positive for Annexin V and negative for propidium iodide (PI); (**G**) Proportions (%) of sub-G1 cells in the cell cycle distribution. Panels (D), (F) and (G) show the results at 72 h after transfection with si-control or si-FGFR2s. *P < 0.05.

Given the significant relationship between FGFR2 IHC positivity and tumor depth, we hypothesized that FGFR2 expression accelerates tumor cell proliferation. To test this hypothesis, we assayed the cell proliferation by RNA interference. Both of the synthetic siRNAs targeting *FGFR2* effectively suppressed FGFR2 expression in the OACM5.1C cell line (Figure 3A). In this cell line, which overexpresses FGFR2, the siRNA-mediated knockdown of *FGFR2* significantly suppressed the cell proliferation in a time-dependent manner (Figure 3B). Because PI3K-AKT and RAS-ERK are major downstream pathways of FGFR2, de-phosphorylation of AKT and ERK was examined under *FGFR2* knockdown. Compared with the control siRNA, phosphorylated-AKT and phosphorylated-ERK were suppressed by *FGFR2* knockdown in OACM5.1C (Figure 3A). These results suggest that de-phosphorylation of ERK and AKT governs the strong association between FGFR2 expression and tumor cell proliferation. To investigate the underlying mechanism of FGFR2-mediated proliferation, we examined the effect of FGFR2 on cell cycle and apoptosis. According to the cell cycle analysis at 72 h after *FGFR2* knockdown by siRNAs, the FGFR2-overexpressing OACM5.1C tumor cells were significantly accumulated in the G0/G1 phase population (*P* < 0.05), and concomitantly decreased in the G2/M phase population (*P* < 0.05) (Figure 3C, 3D). At the same time point, the number of apoptotic cells had significantly increased (*P* < 0.05, Figure 3E, 3F), thereby increasing the sub-G1 phase population (*P* < 0.05, Figure 3G).

### Proliferation, cell cycle and apoptosis assays of FGFR2-stable transfected cell line

**Figure 4:**
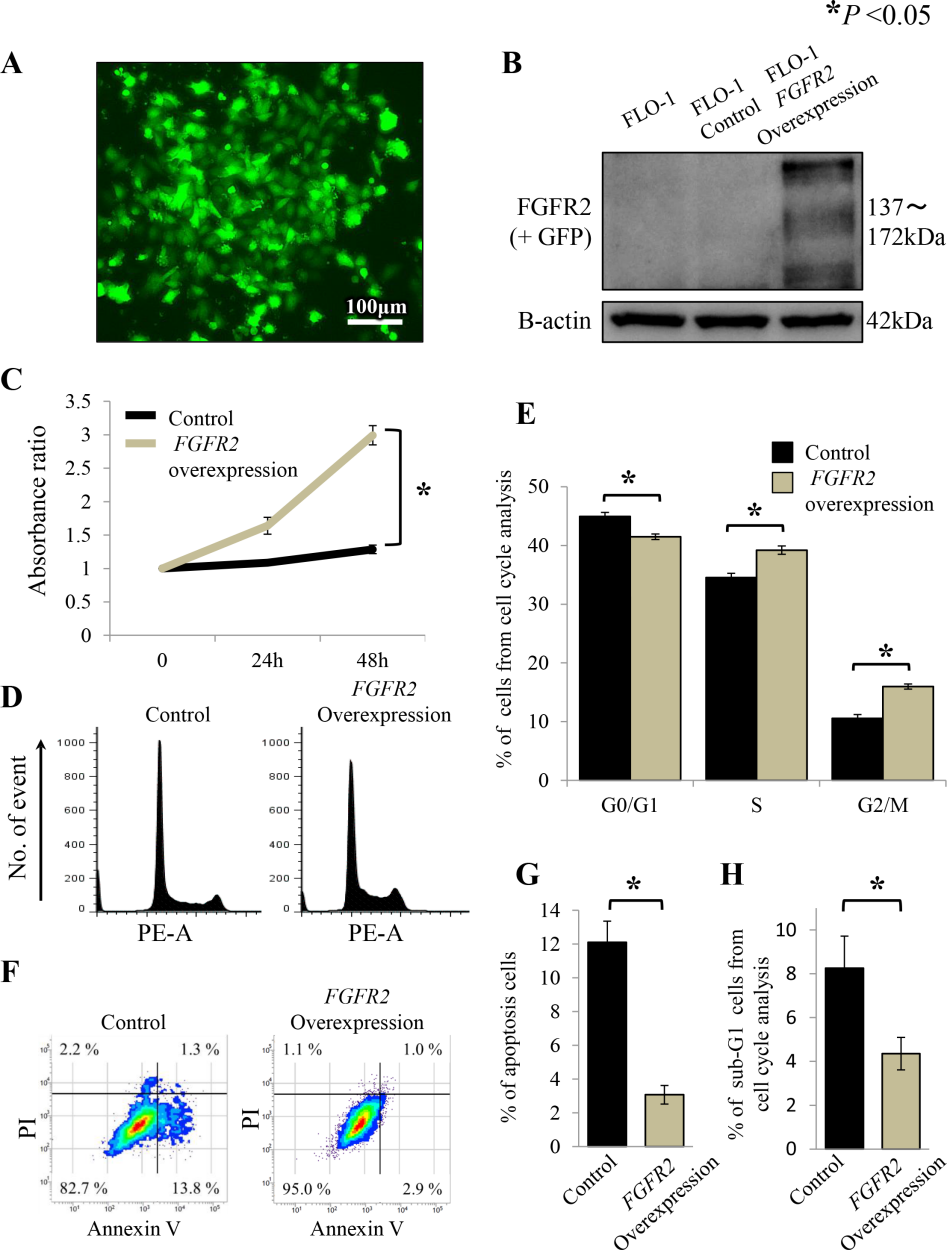
FGFR2 overexpression promotes cell proliferation through cell cycle progression and anti-apoptosis in FLO-1 cells stably transfected with FGFR2 (**A**) FLO-1 cells stably transfected with FGFR2 were confirmed by GFP expression; (**B**) FGFR2 overexpression was confirmed by western blot analysis; (**C**) Cell proliferation of FGFR2 overexpressing cells; (**D**) Distributions of cell cycle populations; (**E**) Proportions (%) of G0/G1, S, and G2/M in the cell cycle distribution; (**F**) Distributions of apoptotic cells; (**G**) Apoptotic cells, identified as positive for Annexin V and negative for propidium iodide (PI); (**H**) Proportions (%) of sub-G1 cells in the cell cycle distribution. *P < 0.05.

To examine the effects of FGFR2 overexpression, *FGFR2IIIb-AcGFP1* coding vectors were transfected into the FLO-1 cell line, which neither amplifies *FGFR2* nor expresses FGFR2. *FGFR2*-stable transfectants were validated by their GFP expression (Figure 4A). FGFR2 overexpression was confirmed by western blot analysis (Figure 4B).

In the proliferation assay, *FGFR2*-overexpressing FLO-1 cells exhibited significantly higher growth over time than cells not transfected with *FGFR2* (*P* < 0.05, Figure 4C). Moreover, in the cell cycle analysis, the G0/G1 phase population of the *FGFR2*-overexpressing cells was significantly decreased, while the G2/M phase population significantly accumulated (*P* < 0.05, Figure 4D, 4E), relative to the non-transfected control cells. FGFR2 overexpression was also inversely associated with cell apoptosis (*P* < 0.05, Figure 4F, 4G), thereby decreasing the sub-G1 phase population (*P* < 0.05, Figure 4H).

### Anti-proliferative effects of a pan-FGFR inhibitor AZD4547

We next investigated whether pan-FGFR inhibitor AZD4547 can therapeutically target the EGJ adenocarcinoma cell line OACM5.1C, which overexpresses FGFR2. For this purpose, we altered the phosphorylated-AKT and phosphorylated-ERK expressions in OACM5.1C cells, and assayed the cell proliferation. Because OACM5.1C cells express low basal levels of phosphorylated-AKT (Figure 3A), these assays were also performed under FGFR2 activation stimulated by FGF7.

**Figure 5:**
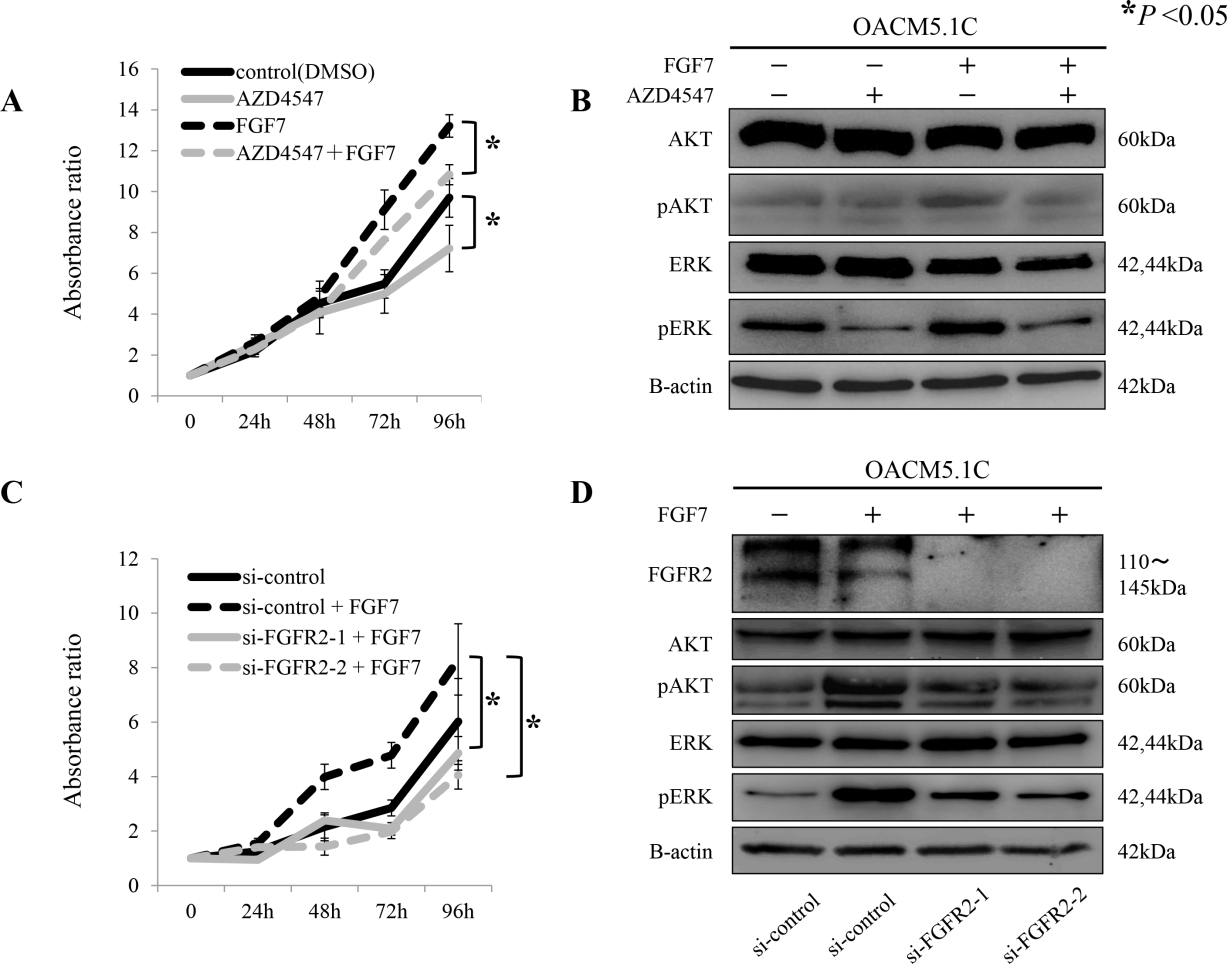
Proliferative inhibition of tumor cells by the pan-FGFR inhibitor AZD4547, and by siRNAs targeting *FGFR2*, through de-phosphorylation of AKT and ERK (**A**) Proliferation assay of tumor cells exposed to AZD4547 with and without FGF7 stimulation; (**B**) De-phosphorylation of AKT and ERK by AZD4547, with and without FGF7 stimulation; (**C**) Proliferation assay using siRNAs targeted against *FGFR2* (si-FGFR2), with and without FGF7 stimulation; (**D**) De-phosphorylation of AKT and ERK after FGFR2 knockdown by si-FGFR2, with and without FGF7 stimulation. *P < 0.05.

In the proliferation assay, AZD4547 significantly suppressed the tumor cell growth (*P* < 0.05, Figure 5A). FGF7 stimulation yielded a similar result (*P* < 0.05, Figure 5A). AZD4547 treatment clearly de-phosphorylated the ERK, regardless of FGF7 stimulation. However, the change in the de-phosphorylated AKT was more evident under FGF7 stimulation than under no stimulation (Figure 5B). Importantly, selective inhibition of FGFR2 by siRNAs yielded similar results (Figure 5C, 5D).

## DISCUSSION

This study examined the relationship between *FGFR2* amplification and FGFR2 expression. *FGFR2* copy number was amplified in 21 (15%) of the 140 assayed EGJ adenocarcinoma cases, and FGFR2 expression was elevated in 108 cases (61%) of the 176 cases assayed by IHC. Although *FGFR2* amplification was correlated with FGFR2 expression, only the latter was strongly correlated with tumor depth and patient outcome. Supporting these clinical findings, FGFR2 expression was associated with tumor cell proliferation, and also with cell cycle progression and anti-apoptosis. In addition, the pan-FGFR inhibitor AZD4547 suppressed EGJ adenocarcinoma growth through de-phosphorylation of AKT and ERK. Similar results were obtained in FGFR2 inhibition by a selective siRNA technique. These findings support FGFR2 as a therapeutic target for EGJ adenocarcinoma.

Genomic aberrations of *FGFR2* reportedly lead to tumorigenicity through the FGF-FGFR2 signaling axis, and are strongly correlated with patient outcomes [20, 21]. Although *FGFR2* amplification has been examined in diffuse-type gastric cancer [16, 22], its relation to clinicopathological factors and patient outcomes in EGJ adenocarcinoma has not been clarified. *FGFR2* amplification may play different roles in distal gastric cancer and EGJ adenocarcinoma, as the two cancers exhibit different biology. First, GERD is more closely associated with EGJ adenocarcinoma than with distal gastric cancer. EGJ adenocarcinoma often arises from excessive gastric acid exposure through esophageal reflux, and is not associated with *Helicobacter pylori* infection [23]. Gastric cancer, however, arises in an atrophic gastritis setting, with reduced output of gastric acid. Second, diffuse histology is much less common in EGJ tumors than in distal gastric cancer. Finally, the prognosis is worse in EGJ adenocarcinoma than in distal gastric cancer [24, 25]. Our study found no association between *FGFR2* amplification and histological tumor type or patient outcome in EGJ adenocarcinoma. These results contrast with the reported characteristics of *FGFR2* amplification in gastric cancer.

*FGFR2* amplification has been reported in 4.1–7.2% of gastric cancer cases [16, 22, 26], and in 4.0% of triple-negative breast cancer cases [27]. In the present study, *FGFR2* was amplified in 15% (21/140) of the investigated EGJ adenocarcinoma cases, higher than in previous reports. These differences may have arisen from our use of a single reference (*RNaseP*) in the copy number assay. Nonetheless, in our copy number assay, *FGFR2* amplification status and FGFR2 expression status in the five tested cell lines correlated well with the clinical features of EGJ adenocarcinoma. In addition, our measured PCR-based copy numbers were concordant with FISH [28, 29], and are clinically useful [22, 30, 31].

Although FGFR2 amplification was associated with FGFR2 IHC positivity, FGFR2 IHC positivity, but not FGFR2 amplification, strongly correlated with tumor aggressiveness, and patient outcome in this study. This discrepancy may be explained by the epigenetic or transcriptional regulation of FGFR2 expression. In thyroid cancer, FGFR2 expression is controlled by methylation of the DNA promoter [32]. Some recent reports have shown that microRNAs suppress the expression of RTKs such as HER2 [33]. Specifically, miR125b controls FGFR2 expression in dermatological disease [34]. Considering that miR125b regulates HER2 in gastric cancer [33], we would expect further comprehensive miRNA assays may reveal the underlying mechanism of transcriptional regulation of *FGFR2* in EGJ adenocarcinoma. The regulation of FGFR2 expression in EGJ adenocarcinoma requires further investigation.

In the present study, FGFR2 expression was strongly associated with the depth of invading tumor and with poor outcome. Paterson et al. reported the prevalence of FGFR2 overexpression in esophageal adenocarcinoma as 34% (124/367 cases). They found no association between FGFR2 overexpression and patient outcome [35]. Moreover, their proportion of FGFR2 overexpression was far less than ours (34% vs. 61%), although both studies used the same antibody. We attribute this discrepancy to the intra-tumor heterogeneity of FGFR2 expression. Whereas we stained cross-sections in the FGFR2 IHC assay, Paterson et al. stained tissue arrays (diameter 0.6 mm). We suspect that cross-sections more likely represent the overall FGFR2 expression status than tissue array.

According to our experimental data, FGFR2-expressing EGJ adenocarcinoma cell lines were sensitive to a pan-FGFR inhibitor of AZD4547. These results were exactly reproduced by selective FGFR2 inhibition by siRNAs. In EGJ adenocarcinoma, the FGF-FGFR2 signaling axis may be a crucial pathway in tumor progression; thus, this cancer may be adequately managed by an FGFR2-specific inhibitor. Moreover, FGFR2 expression may be a better biomarker of tumor aggressiveness in EGJ adenocarcinoma than *FGFR2* amplification. To the best of our knowledge, there is no clinical study examining FGFR-inhibitor focusing on the patients with EGJ adenocarcinoma alone. A recent phase II clinical trial of SHINE study compared AZD 4547 with paclitaxel in previously treated cases of advanced gastric or EGJ cancer. In their study, despite that *FGFR2* amplification status has been used to select likely responders to AZD4547 treatment, the clinical responses were not impressive [36]. One possible explanation may be their methodology for *FGFR2* amplification, and FGFR2 expression testing. *FGFR2* amplification status was 9%, which was confirmed by FISH testing. In those *FGFR2* amplified case, FGFR2 expression was observed in only 21%, which was much lower than 76.2% (16/21 cases) that we observed in our study. Further study would be needed for more adequate assays for *FGFR2* amplification or FGFR2 expression.

Our study is limited by its retrospective design. Nonetheless, our present experimental results convincingly support the clinical findings. The role of oncogenic amplification in esophageal or EGJ adenocarcinoma has recently received much attention [12–15]. This study focused on the mechanistic role of FGFR2 amplification in EGJ adenocarcinoma. To confirm our findings, we require evidence from large-scale prospective studies.

In conclusion, we found that (i) *FGFR2* amplification is associated with FGFR2 expression, and (ii) FGFR2 expression, but not *FGFR2* amplification, is associated with depth of tumor invasion and poorer outcomes in EGJ adenocarcinoma. Our data emphasize that FGFR2 can be a therapeutic target for EGJ adenocarcinoma.

## MATERIALS AND METHODS

### Cell lines and culture

Five EGJ adenocarcinoma cell lines, OACM5.1C, OE19, OE33, SK-GT-4 and FLO-1, were purchased from the European Collection of Cell Cultures (ECACC; Salisbury, UK). FLO-1 was cultured in DMEM (Sigma-Aldrich, St Louis, MO, USA) containing 10% fetal bovine serum (FBS) in a 5% CO_2_ air-humidified atmosphere at 37°C. The other cell lines (OACM5.1C, OE19, OE33 and SK-GT-4) were cultured in RPMI-1640 (Life Technologies, Carlsbad, CA, USA) under the same conditions, according to the manufacturer's recommendations.

### Patients and study design

The study subjects were 176 consecutive chemotherapy-naive patients with EGJ adenocarcinoma (Siewert type I-III), whose formalin fixed paraffin-embedded (FFPE) archival tissues were available. These patients were treated at Kumamoto University Hospital (Kumamoto, Japan) from February 2000 to March 2014 (*N* = 112), or at Kyushu University Hospital (Fukuoka, Japan) from April 2005 to February 2014 (*N* = 64). There were 139 (139/176 = 79%) men and 37 (37/176 = 21%) women. The median age of the patients was 69 (range 36-89 years). Surgical resection and endoscopic submucosal dissection (ESD) was performed in 168 (168/176 = 96%) and eight (8/176 = 4%) cases, respectively. Of the 168 surgically resected cases, 152 (152/168 = 90%) underwent surgery with curative intent, and 16 (16/168 = 10%) with palliative intent. Treatment dates were retrospectively obtained from the patients’ records. Patients were observed until death or 30 January 2015, whichever came first. The mean follow-up time in the survival analysis dataset was 32.1 months (range 1–117 months). Disease staging was based on the Union for International Cancer Control classification (7th edition) of esophageal cancer. Use of the clinical data was approved by the human ethics review committee of the Graduate School of Medicine, Kumamoto University and by Kyushu University (Institutional Review Board numbers; 858 for Kumamoto University and 27–27 for Kyushu University).

### DNA extraction

FFPE tissue specimens were collected from each resected case, and the tumor DNA was extracted. Tumor lesions on hematoxylin and eosin-stained slides without coverslips were marked by a pathologist (YI). Each marked area was scraped by the macro-dissection method, and tissue DNA was isolated using an FFPE Kit (Qiagen, Hilden, Germany) [37]. In 31 of the 176 cases, the tumors were too small to be extracted. Finally, tumor DNA was successfully isolated from 145 cases (145/176 = 82%, Supplementary Figure 1). DNA extraction from the cell lines was performed with a QIAmp DNA Mini Kit (Qiagen). The DNA concentration was measured by NanoDrop2000 (Thermo Scientific, Waltham, MA, USA).

### Copy number assay for FGFR2

Copy number assay was performed by a real-time PCR reaction. The copy number of *FGFR2* was quantified relative to a reference assay using *ribonuclease P* (*RNase P*) as previously reported [22,30,31]. Tumor DNA was analyzed with TaqMan Copy Number Assays (Life Technologies) using Hs05182482_cn (intron 14 of *FGFR2*, amplicon length 80 bp; Life Technologies) as primers. Similarly, the copy number of *RNaseP* was assayed by TaqMan Copy Number Reference Assays (Life Technologies) [38]. Real-time PCR reaction was performed by the 7900HT Fast Real-Time PCR System (Life Technologies). Into each well, we deposited 5 μl of TaqMan genotyping master mix, 10 ng of genomic DNA and 0.5 μl of each primer (total volume = 10 μl). The PCR conditions consisted of initial denaturing at 95°C for 10 min, 40 cycles of 95°C for 15 s and 60°C for 1 min. The data were analyzed by the software packages SDS 2.4 (Life Technologies) and Copy Caller (Life Technologies).

### Fluorescence *in situ* hybridization

Dual-color FISH analysis was performed on formalin-fixed paraffin-embedded (FFPE) tissue. After deparaffinization and dehydration, the sections of FFPE tissue were digested in 0.1 N HCl for 8–12 min, and then washed in phosphate-buffered saline (PBS) for 5 min at room temperature. FISH *FGFR2* probe was labeled with bacterial artificial chromosomes (BACs), RP11-7P17, RP11-984I17, RP11-78A18, RP11-615K11 and RP11-62L18, which were labeled with Cy3 (Chromosomescience laboratory, Sapporo, Japan). FISH *chromosome 10 centromere* (*CEN10*) was labeled with BACs RP11-300L24, RP11-178A10, RP11-110L24, and RP11-379D20, which were labeled with FITC (Chromosomescience laboratory, Sapporo, Japan). After dehydration and dry up, each FISH probe was applied to each targeted area, and then the slides were sealed with coverslips. The section was denatured at 90°C for 10 min, followed by overnight hybridization at 37°C in a wet chamber. Hybridized slides were washed in 2x saline-sodium citrate buffer (SSC), and then coverslips were removed gently. The slides were stringently washed in 50% formamide/2x SSC for 20 min at 37°C, and the kept in 1× SSC for 15 min at room temperature. The slides were incubated with anti-FITC-Alexa488 in blocking reagent for 60 min at 37°C. After post-hybridization washing, the slides were counterstained with 4′,6-diamidino-2-phenylindole (DAPI). The FISH images were captured with a fluorescence microscope (BZ-X710, Keyence, Japan).

### Quantitative real-time reverse transcription-polymerase chain reaction

The *FGFR2*-mRNA expression level was measured in triplicate and calculated as the fold change relative to a reference gene using the TaqMan (Life Technologies) primer-probe sets, according to the manufacturer's protocol. Results were normalized by the relative expression levels of hypoxanthine phosphoribosyltransferase 1 (HPRT-1). All quantitative real-time reverse transcription-polymerase chain reactions (qRT-PCRs) for mRNA were run on the Light Cycler 480 System II (Roche Diagnostics, Basel, Switzerland). The relative amounts of genes were measured by the 2^−ΔΔCT^ method. The sequences of the primers used in this study are listed in Supplementary Table 1.

### Western blot analysis

Each protein sample was subjected to sodium dodecyl sulfate-polyacrylamide gel electrophoresis, transferred to a nitrocellulose membrane, and exposed to primary antibodies. Signals were detected by incubation with secondary antibodies labeled using the ECL Detection System (GE Healthcare, Little Chalfont, UK). The primary antibodies, FGFR2 (#11835s), AKT (#9272), phospho-AKT Ser473 (#9271), ERK1/2 (#9102), phospho-ERK1/2 Thr202/Tyr204 (#4376) and Β-actin (#4967s), were purchased from Cell Signaling Technology (Danvers, MA, USA).

### Immunohistochemical staining of FGFR2 expression

Immunohistochemical (IHC) staining was performed on 4-μm FFPE sections. Each section was autoclaved in 10 mmol/L citrate buffer (pH 6.0) for 15 min, and endogenous peroxidase activity was blocked by adding 3% hydrogen peroxide for 5 min. The sections were then incubated overnight with 1:1000 (in 0.1 mol/L phosphate-buffered saline) diluted primary mouse monoclonal anti-FGFR2 antibody (ab58201, Abcam, Cambridge, UK) at 4°C, followed by incubation with a biotin-free horseradish peroxidase-labeled polymer (Envision Plus detection system; Dako, Glostrup, Denmark) for 1 h at room temperature. The reaction was visualized by diaminobenzidine solution and hematoxylin counterstaining. Two pathologists (YI and YN, who were blinded to the other data) recorded the membranous and cytoplasmic FGFR2 expression in the cancer cell, scoring absent, faint, moderate, or strong staining, respectively. FGFR2-IHC positive was assigned for the cases with moderate-strong staining, and negative for those with absent or faint staining, which was the same as previously reported [35]. The value of kappa was 0.91 (*P* < 0.001), indicating almost perfect agreement. For positive cases, we also scored percentage of positivity in the tumor. The concordance between the two observers was measured using kappa statistics.

### Chemical compounds

A pan-FGFR inhibitor AZD4547 was purchased from AdooQ BioScience (Irvine, Carlsbad, CA, USA). For *in vitro* experiments, AZD4547 was prepared in dimethyl sulfoxide (DMSO). FGF7 was purchased from Wako (Osaka, Japan).

### Small-interfering RNAs-mediated FGFR2 depletion

Two individual *FGFR2*-specific small-interfering RNAs (siRNAs) were chemically synthesized to target different regions of *FGFR2* (s5174 and s5175, Life Technologies). As the negative control, we used a silencer select RNAi negative control (Life Technologies). Cells were seeded at 1 × 10^6^ cells in a electroporation chamber (CUY 532, 3 mm × 10 mm × 5 mm; Nepagene, Chiba, Japan), and 100 μl of siRNA (10 nmol/L, diluted by Opti-MEM) was added. Electroporation was performed by applying two square pulses (175 V) of 5 ms duration. The pulses were separated by a 50-ms interval, and provided by a pulse generator NEPA21-S (Nepagene), as described previously [39, 40]. After 6 h, the medium for the electroporated cells was replaced with fresh medium. At 72 h after electroporation, the cells were harvested and used in the following assays.

### Construction of stable FGFR2 transfectant

The cDNA clone encoding full-length human FGFR2IIIb was obtained using a gene-specific RT primer and a PCR primer from the RNA of MCF7. FGFR2IIIb cDNAs were subcloned into the modified pIRESpuro3 vector containing the AcGFP1 coding sequence at the cut sites Nhe I and Xho I. All sequences were confirmed by direct sequencing. The FGFR2IIIb-AcGFP1 lesion was transfected to the targeted cell by electroporation. The stable transfectants were selected using 1 μg/ml puromycin.

### Cell proliferation assay

Cells were seeded at 3000 cells per well in a 96-well plate, and provided with fresh medium each day. After treatment with siRNA or chemicals, the number of viable cells was counted using a Cell Counting Kit-8 (CCK-8; Dojin Laboratories, Kumamoto, Japan) and an automatic microplate reader (Molecular Devices, Sunnyvale, CA, USA) at five time-points: 0, 24, 48, 72, and 96 h after seeding, following the manufacturers’ instructions.

### Apoptosis assay, and cell cycle analysis by flow cytometry

Transfected cells were seeded at 1 × 10^6^ cells in 100-mm-diameter dishes. After 72 h incubation, the cells were subjected to apoptosis assay and cell cycle analysis. Phosphatidylserine externalization in the apoptosis assay was detected by Annexin V (Millipore, Billerica, MA, USA) and propidium iodide (PI) (Sigma-Aldrich) staining. Cells in the early phase of apoptosis stained positive for Annexin V and negative for PI. Cell cycles were analyzed by flow cytometry in a BD FACS Verse flow cytometer (BD Biosciences, San Jose, CA, USA). The cells were diluted to 1 × 10^6^/ml, and fixed by overnight immersion in 70% ethanol at −20°C. Subsequently, the cells were pelleted by centrifugation and re-suspended in phosphate-buffered saline containing 1 μg/ml RNase A (Sigma-Aldrich) and 100 μg/ml PI. The distribution of cells in different phases of the cell cycle was calculated by FlowJo software (TOMY Digital Biology, Tokyo, Japan).

### Statistical analysis

Statistical analyses were performed by JMP version 10 (SAS Institute Inc., Cary, NC, USA) and Excel 2010 (Microsoft, Redmond, WA, USA) software. All of the above experiments were triplicated, and the continuous data are shown as the mean ± standard deviation (SD). The associations between clinicopathological factors and FGFR2 amplification status (negative vs. positive) or FGFR2 IHC (negative vs. positive) were identified in univariate analyses. Categorical data (sex, tumor location by Siewert classification, tumor depth, lymph node metastasis, distant metastasis, and histopathological types) were analyzed by *χ* ^2^ test (case number ≥ 5) or Fisher's exact test (case number < 5); continuous data (age at resection, and tumor size) were analyzed by Student's *t*-test. The relationships between clinicopathological factors and FGFR2 expression were assessed by a multivariate logistic regression model. We initially included age (≥ 70 vs. <70), sex (male vs. female), tumor location by Siewert classification (I–II vs. III), tumor depth (T2–T4 vs. T1), lymph node metastasis (positive vs. negative), distant metastasis (positive vs. negative), and histopathological types (poorly moderated vs. well moderate). Survival analysis related to FGFR2 amplification or FGFR2 IHC status was performed by the Kaplan-Meier method and log-rank test.

The correlation between *FGFR2* amplification status and FGFR2 IHC status was considered significant at the *P* = 0.05 level. The association between FGFR2 amplification/FGFR2 IHC status and two kinds of patient prognosis [cancer-specific survival (CSS), and overall survival (OS)] was considered significant at the *P* = 0.0125 level (= 0.05/4). During multiple hypothesis testing of the associations between FGFR2 amplification/FGFR2 IHC status and the remaining seven clinicopathological factors, we adjusted the significant *P* value to 0.004 (0.05/14) by Bonferroni correction.

## SUPPLEMENTARY MATERIALS FIGURES AND TABLES



## Abbreviations

EGJ: esophagogastric junction
GERD: gastro-esophageal reflux disease
FGFR2: fibroblast growth factor receptor 2
FGF: fibroblast growth factor
FISH: fluorescence in situ hybridization
FFPE: formalin fixed paraffin embedded
ESD: endoscopic submucosal dissection
RNase P: ribonuclease P
qRT-PCR: quantitative real-time reverse transcription-polymerase chain reaction
HPRT-1: hypoxanthine phosphoribosyltransferase 1
IHC: immunohistochemical
DMSO: dimethyl sulfoxide
siRNA: small-interfering RNAs
PI: propidium iodide
SD: standard deviation
CSS: cancer-specific survival
OS: overall survival.
