# Supplementary material for: Fibroblast growth factor receptor 2 expression, but not its genetic amplification, is associated with tumor growth and worse survival in esophagogastric junction adenocarcinoma
Source: Oncotarget. 2016 Feb 27;7(15):19748–61. doi: 10.18632/oncotarget.7782 (PMC4991416; doi:10.18632/oncotarget.7782)
Supplement: Supplementary file 1 [file oncotarget-07-19748-s001.pdf]

Fibroblast growth factor receptor 2 expression, but not its genetic amplification, is associated with tumor growth and worse survival in esophagogastric junction adenocarcinoma

Supplementary Materials

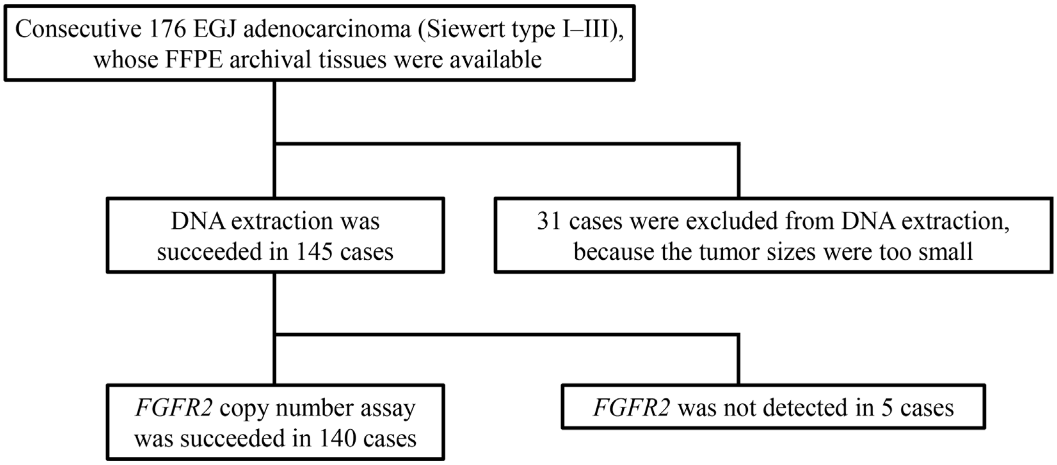

Supplementary Figure S1: Flow chart showing the inclusion/exclusion criteria for copy number assay and immunohistochemical staining.

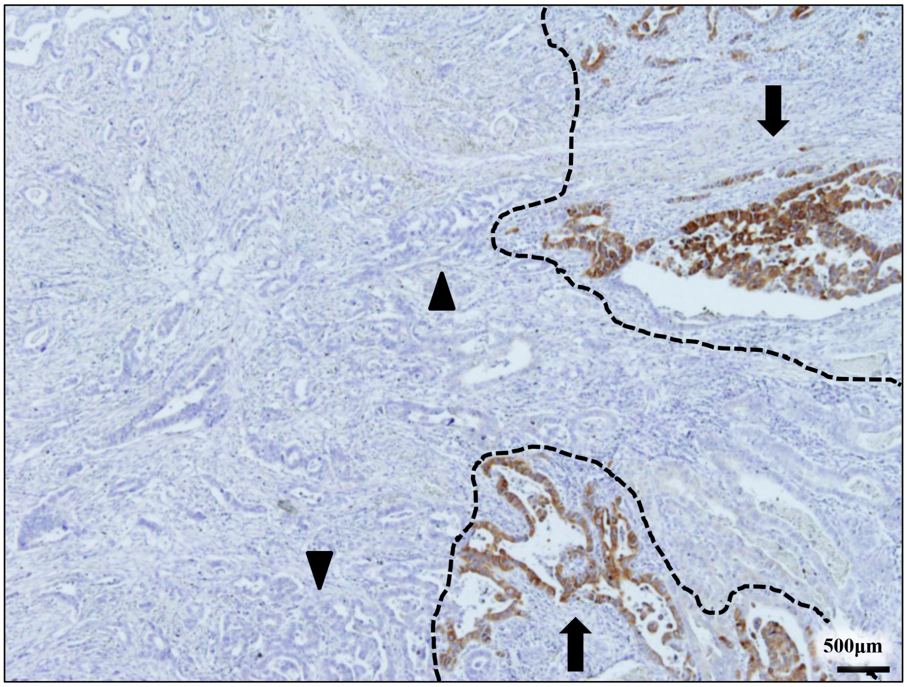

Supplementary Figure S2: Heterogeneous FGFR2 expression in EGJ adenocarcinoma. The tumor cells area surrounded by dotted lines are negative for FGFR2 expression. The arrows show FGFR2 positive cancer cells. The arrowheads show FGFR2 negative cancer cells.

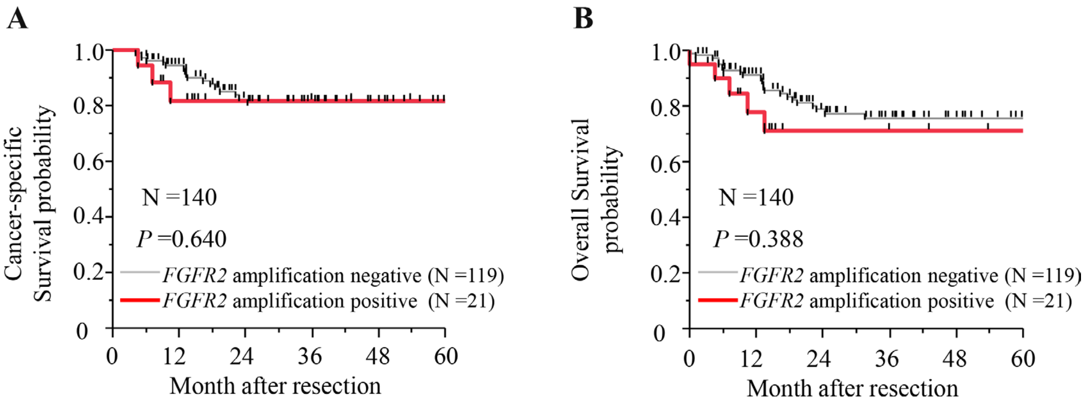

Supplementary Figure S3: Patient outcomes in cases of positive (red) and negative (gray) *FGFR2* amplification (*N* = 140). (**A**) cancer-specific survival; (**B**) overall survival.

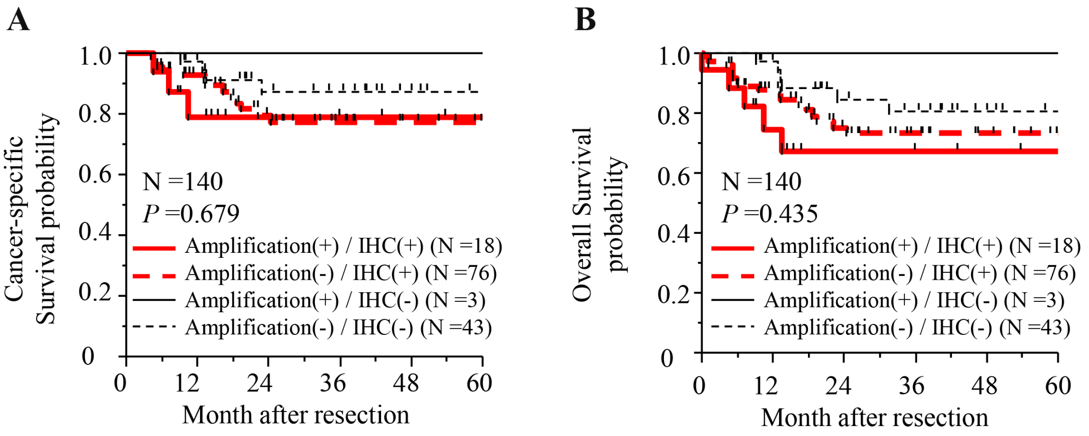

Supplementary Figure S4: Patient outcomes according to combinations of *FGFR2* amplification and FGFR2 IHC status (*N* =140). (**A**) cancer-specific survival; (**B**) overall survival.

Supplementary Table S1: Primer sequences used in qRT–PCR of mRNA

Gene	Primer sequence
FGFR2	F 5′-GGACCCAAAATGGGAGTTTC-3′
	R 5′-ACCACTTGCCCAAAGCAA-3′
HPRT-1	F 5′-TGACCTTGATTTATTTTGCATACC-3′
	R 5′-CGAGCAAGACGTTCAGTCCT-3′

Supplementary Table S2: Univariate and multivariate logistic analysis of *FGFR2* amplification status in EGJ adenocarcinoma patients with tumor resection

Factors		***FGFR2* amplification positive status (%)**	Univariate analysis			Multivariate analysis		
			**OR**	**95% CI**	***P* value**	**OR**	**95% CI**	***P* value**
**Age**	≥ 70/< 70	9 (14%)/12 (16%)	0.82	0.31–2.07	0.669	0.71	0.25–1.95	0.511
**Sex**	Male/female	17 (15%)/4 (15%)	1.02	0.34–3.79	0.976	1.21	0.31–3.93	0.760
**Siewert classification**	I–II/III	13 (15%)/8 (15%)	1.01	0.37–2.59	0.981	1.02	0.38–2.85	0.971
**Tumor depth**	T2–T4/T1	15 (15%)/6 (14%)	1.13	0.42–3.37	0.816	1.08	0.31–3.94	0.903
**Lymph node metastasis**	Positive/negative	12 (18%)/9 (12%)	1.66	0.65–4.35	0.286	1.90	0.63–6.13	0.256
**Distant metastasis**	Positive/negative	2 (15%)/19 (15%)	1.03	0.15–4.26	0.968	1.04	0.22–7.58	0.961
**Histopathological type**	Poorly/well-moderate	4 (9%)/17 (18%)	0.48	0.13–1.41	0.192	0.38	0.10–1.21	0.104

In multiple-hypothesis testing, the significant *P*-value was adjusted to $P = 0.05/14 = 0.004$. Thus, a *P* value between 0.05 and 0.004 should be regarded as borderline significant.
CI, confidence interval; *EGJ,* esophagogastric junction; *FGFR2,* fibroblast growth factor receptor *2*; *OR,* odds ratio
$N = 176$.
